# Spinal Endoscopy for Delayed-Onset Lumbar Radiculopathy Resulting from Foraminal Stenosis after Osteoporotic Vertebral Fracture: A Case Report of a New Surgical Strategy

**DOI:** 10.1155/2018/1593021

**Published:** 2018-10-25

**Authors:** Yuyu Ishimoto, Hiroshi Yamada, Elizabeth Curtis, Cyrus Cooper, Hiroshi Hashizume, Akihito Minamide, Yukihiro Nakagawa, Munehito Yoshida

**Affiliations:** ^1^Department of Orthopedic Surgery, Wakayama Medical University, Japan; ^2^MRC Lifecourse Epidemiology Unit, University of Southampton, Southampton, Hampshire, UK; ^3^Arthritis Research UK/MRC Centre for Musculoskeletal Work and Health, Southampton General Hospital, Southampton, Hampshire, UK

## Abstract

There is little evidence regarding the optimal approach to treatment for delayed-onset lumbar radiculopathy due to foraminal stenosis after osteoporotic vertebral fracture. Here, we describe the use of spinal endoscopy for the treatment of this disabling condition, in an 80-year-old woman presenting with severe radicular pain following an osteoporotic lumbar compression fracture. Radiographic findings showed the compression of the L2 root within the foramen, and computed tomography identified a fragment of the posterior wall of the vertebral body under the pedicle. Since the patient had little back pain and was relatively frail, we decided to perform foraminal decompression via a lateral approach using spinal endoscopy. Intraoperative findings demonstrated degenerative changes as well as a fragment of the posterior wall of the fractured vertebral body in the foramen, covered in a fibrous film. The nerve was decompressed on removal of the fragment. After surgery, the patient experienced immediate pain relief. The preoperative Japanese Orthopedic Association (JOA) and visual analogue scale (VAS) scores were 9 and 82, respectively, and at 36-month follow-up, scores were reduced to 19 and 34, respectively.

## 1. Introduction

Osteoporotic spinal fractures are a major public health issue and are occurring at an increasing rate, causing significant disability in the elderly population [1, 2]. There are various reports of spinal paralysis as a result of these fractures, and the treatment for this condition is now well established [3, 4].

Here, we present the case of a patient who suffered from severe radiculopathy due to foraminal stenosis after osteoporotic compression fracture. Foraminal stenosis is commonly seen in the elderly as a result of degenerative changes [5, 6]; in this case, further foraminal stenosis was induced after an osteoporotic spinal fracture. The patient's symptoms were similar to those of lumbar spinal stenosis, with exacerbation of her symptoms on standing and walking. Despite this patient having a vertebral fracture with nonunion, she suffered from leg pain rather than low back pain. In addition, her lumbar MRI showed mild central stenosis without protrusion of the vertebral posterior wall. Following clinical examination and review of the imaging, we judged that the lesion was in the foramen, so we operated using spinal endoscopy, finding foraminal bony fragments which could be removed on minimally invasive surgery. As vertebral fractures are common, there may be many patients similar to ours who may benefit from a minimally invasive endoscopic to remove bony fragments; however, to our knowledge, little is known about the effectiveness of such an approach.

Our intraoperative findings clarified the cause of her symptoms, and after surgery, the patient has remained almost pain-free. We propose that our surgical strategy using spinal endoscopy is an effective treatment option for frail patients, including those with severe osteoporosis.

## 2. Case Presentation

**Figure 1:**
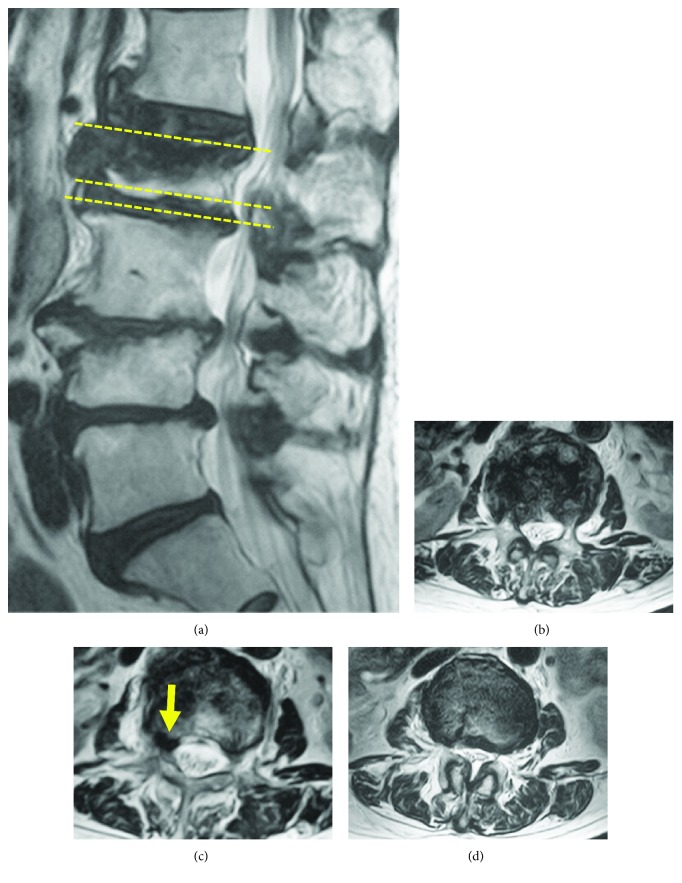
(a) Preoperative sagittal MR image showing low intensity zone in upper L2 vertebral body. (b), (c), and (d): mild canal stenosis at the L2 level and moderate stenosis at the L2/3 level. There was a mass in the rt L2/3 foramen (arrow in (c)).

**Figure 2:**
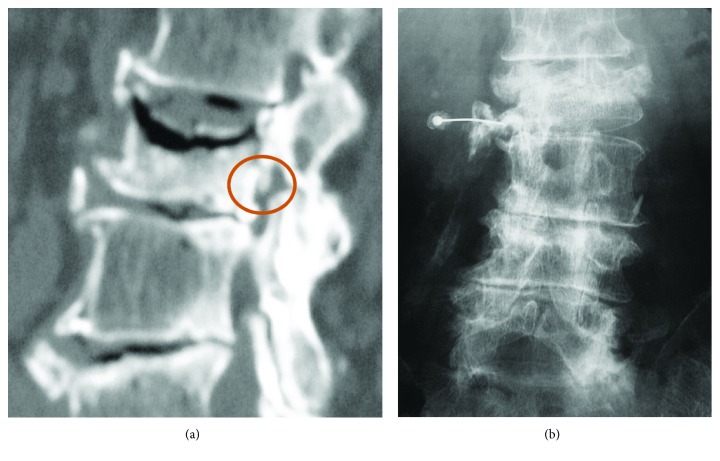
(a, b) Three-dimensional computed tomography image showing the bone tip under the right L2 pedicle. Left L2 root block was effective temporally, and radiography showed that left L2 root compressed at intervertebral foramen.

An 80-year-old woman fell in her bathroom at home and experienced acute-onset low back pain. Following a plain radiograph, she was diagnosed with a L2 compression fracture and began conservative treatment. One month after the injury, she began experiencing severe radicular pain when walking, with no obvious precipitant. After 3 months of treatment, she visited our university hospital as the cause of her radicular pain was still unclear. On lying supine, she had no pain, but when she stood up or walked, she experienced severe pain in the inside of her thigh in addition to mild lower back pain. Magnetic resonance imaging showed a change in the signal intensity within the L2 vertebral body (Figure 1(a)), but little canal stenosis at the L2, and L2/3 levels (Figures 1(b) and 1(c)). Computed tomography demonstrated a bone tip under the pedicle (Figure 2(a)). A left L2 root block was effective in reducing her pain temporarily. Radiography demonstrated compression of the L2 root in the foramen (Figure 2(b)). In the case like this with nonunion, fusion surgery is usually undergone. The patient were very old and with poor condition for surgery; further, the patient had little low back pain. We explained the risk without fusion surgery to the patient and attempted to decompress the L2 root using spinal endoscopy.

The patient was able to walk the day after surgery. No complications related to surgery occurred perioperatively, and her pain was relieved immediately. Her preoperative Japanese Orthopedic Association (0–29) and visual analog scale (0–100) scores were 9 and 82, respectively, and at the 36-month follow-up, scores changed to 19 and 34, respectively.

## 3. Surgical Procedure

**Figure 3:**
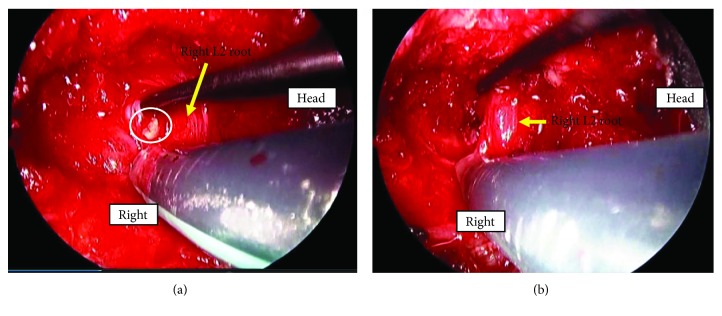
(a) There was the fragment covered in a fibrous film of the posterior wall of fractured vertebral body compressing L2 root from the front at foramen. (b) After removing the fragment, the root was decompressed.

After securing informed consent from the patient and explaining to her family regarding the procedure and any possible complications, we performed endoscopic spinal surgery. We used the Minimal Exposure Tubular Retractor System (MET-Rx®: Medtronic Sofamor Danek, Memphis, TN, USA) via a lateral approach [7]. After expanding the intervertebral foramen with little sacrifice of the facet, we removed a fragment covered in a fibrous film from the posterior wall, under the pedicle (Figures 3(a) and 3(b)). The fragment was in the foramen independently, and we hypothesized that it became nonunion and wrapped with soft tissue. We decompressed with little sacrifice of the facet and with minimal blood loss.

## 4. Discussion

We report a new surgical strategy using spinal endoscopy, performed in a patient with delayed-onset lumbar radiculopathy due to foraminal stenosis after osteoporotic spinal fracture. The patient's radicular pain was relieved immediately following surgery, and no complications occurred in the perioperative period. Degenerative changes were found intraoperatively, including superior facet hypertrophy and bulging of the disc, as well as a fragment of the vertebral posterior wall, covered in a fibrous film, in the foramen. In addition, we think that further foraminal stenosis occurred due to the instability under pedicle in L2 vertebrae because of nonunion when the patient stood.

The most widely used surgical approaches to osteoporotic spinal fractures are fusion surgery including vertebroplasty and kyphoplasty [8]. For foraminal stenosis, decompression surgery is commonly used, via facetectomy and elevation of the disc height by interbody fusion [9]. However, fusion surgeries can result in a variety of complications such as blood loss, infection, and pseudarthrosis [10–12], which are particularly common in postmenopausal women [10]. In addition, Bogdanffy et al. [13] reported that the bone mineral density of the vertebral body above fused segments can decrease significantly. Therefore, fusion strategies such as vertebroplasty and kyphoplasty for patients with severe osteoporosis can lead to further vertebral fractures, possibly leading to further foraminal stenosis as a result of increased pressure in the vertebral body. Hence, decompressing whilst visualizing the nerve endoscopically may be appropriate in frail patients.

Further reports of this surgical approach are required to justify its use, particularly in the elderly, who are prone to complications. We believe that minimally invasive endoscopic surgery may be an attractive option for many frail patients with vertebral fractures in whom high-risk fusion surgery is the only alternative.

## 5. Conclusion

Spinal endoscopy was performed in a patient with delayed-onset lumbar radiculopathy due to foraminal stenosis following an osteoporotic spinal fracture. The surgery effectively relieved the patient's pain, and no perioperative complications occurred. We propose that endoscopic surgery is a feasible and minimally invasive strategy for treating this condition and would be particularly suitable for frail patients at the highest risk of anaesthetic, operative, and postoperative complications.
